# Mechanical Ventilation Redistributes Blood to Poorly Ventilated Areas in Experimental Lung Injury*

**DOI:** 10.1097/CCM.0000000000004141

**Published:** 2020-02-13

**Authors:** John N. Cronin, Douglas C. Crockett, Andrew D. Farmery, Göran Hedenstierna, Anders Larsson, Luigi Camporota, Federico Formenti

**Affiliations:** 1 Centre for Human and Applied Physiological Sciences, Faculty of Life Sciences and Medicine, King’s College London, London, United Kingdom.; 2 Nuffield Division of Anaesthetics, Nuffield Department of Clinical Neurosciences, University of Oxford, Oxford, United Kingdom.; 3 Hedenstierna Laboratory, Department of Medical Sciences, Uppsala University, Uppsala, Sweden.; 4 Hedenstierna Laboratory, Department of Surgical Sciences, Uppsala University, Uppsala, Sweden.; 5 Department of Adult Critical Care, St Thomas’ Hospital, Guy’s and St Thomas’ NHS Foundation Trust, King’s Health Partners, London, United Kingdom.

**Keywords:** pulmonary circulation, respiratory distress syndrome, adult, tomography, x-ray computed, ventilation-perfusion ratio, ventilator-induced lung injury, swine

## Abstract

Determine the intra-tidal regional gas and blood volume distributions at different levels of atelectasis in experimental lung injury. Test the hypotheses that pulmonary aeration and blood volume matching is reduced during inspiration in the setting of minimal tidal recruitment/derecruitment and that this mismatching is an important determinant of hypoxemia.

**Design:**

Preclinical study.

**Setting:**

Research laboratory.

**Subjects:**

Seven anesthetized pigs 28.7 kg (sd, 2.1 kg).

**Interventions:**

All animals received a saline-lavage surfactant depletion lung injury model. Positive end-expiratory pressure was varied between 0 and 20 cm H_2_O to induce different levels of atelectasis.

**Measurements and Main Results:**

Dynamic dual-energy CT images of a juxtadiaphragmatic slice were obtained, gas and blood volume fractions within three gravitational regions calculated and normalized to lung tissue mass (normalized gas volume and normalized blood volume, respectively). Ventilatory conditions were grouped based upon the fractional atelectatic mass in expiration (< 20%, 20–40%, and ≥ 40%). Tidal recruitment/derecruitment with fractional atelectatic mass in expiration greater than or equal to 40% was less than 7% of lung mass. In this group, inspiration-related increase in normalized gas volume was greater in the nondependent (818 µL/g [95% CI, 729–908 µL/g]) than the dependent region (149 µL/g [120–178 µL/g]). Normalized blood volume decreased in inspiration in the nondependent region (29 µL/g [12–46 µL/g]) and increased in the dependent region (39 µL/g [30–48 µL/g]). Inspiration-related changes in normalized gas volume and normalized blood volume were negatively correlated in fractional atelectatic mass in expiration greater than or equal to 40% and 20–40% groups (*r*^2^ = 0.56 and 0.40), but not in fractional atelectatic mass in expiration less than 20% group (*r*^2^ = 0.01). Both the increase in normalized blood volume in the dependent region and fractional atelectatic mass in expiration negatively correlated with Pao_2_/Fio_2_ ratio (*ρ* = –0.77 and –0.93, respectively).

**Conclusions:**

In experimental atelectasis with minimal tidal recruitment/derecruitment, mechanical inspiratory breaths redistributed blood volume away from well-ventilated areas, worsening Pao_2_/Fio_2_.

## Abstract 2

Supplemental Digital Content is available in the text.

Mechanical ventilation is the mainstay of treatment in the acute respiratory distress syndrome (ARDS) (1) with refractory hypoxemia remaining common (2). The optimal settings for mechanical ventilatory variables, including positive end-expiratory pressure (PEEP), remain difficult to define on the individual patient basis (3). PEEP significantly improves oxygenation in ARDS (4–6) and may mitigate ventilator-induced lung injury (7, 8). Conversely, PEEP reduces cardiac output (9, 10), worsens overdistension injury of well-ventilated regions (3), and may increase mortality (11). Most research aimed at setting the optimum mechanical ventilation variables have focused on alveolar recruitment and lung compliance; fewer studies have investigated the impact of these settings on regional distribution of pulmonary perfusion. Oxygenation is improved by pulmonary ventilation and matching, so should be considered throughout the respiratory cycle when titrating ventilation in ARDS.

Prolonged high inspiratory pressures may worsen oxygenation in patients (12) and in experimental lung injury (13) due to redistribution of blood toward dependent regions. Determining the regional distribution of blood during the time course of a single breath remains challenging. CT scanning is a possible technology; however, a limitation is that a low iodine contrast concentration or a high atelectatic lung density within a voxel can have the same CT number (14–16). When considering the lung, if a voxel has an attenuation of 58 Hounsfield units (HU) with a tube voltage of 140 kVp, it is impossible to determine whether it contains 100% soft tissue or a mixture of 13% gas, 17% soft tissue, and 70% iodinated blood (**Fig. 1*A***, points B and D). Dual-energy CT (DECT) three-material differentiation solves this problem by imaging the same voxel with two different x-ray spectra which will be attenuated to a different degree by each material (Fig. 1, *A* and *B*). By combining it with a dynamic technique imaging a single slice over time (dDECT), it is suited for simultaneously assessing gas and blood volume distributions during the respiratory cycle. Unfortunately, commercial DECT implementations are aimed toward qualitative interpretation of lung parenchymal blood content during apnea, rather than continuous quantification within respiratory cycles.

**Figure 1.**
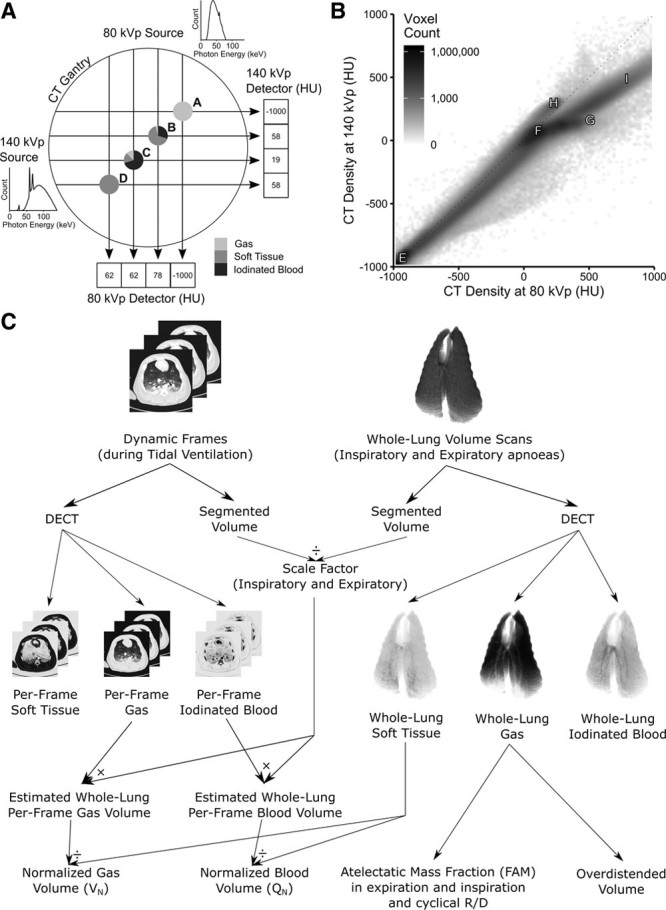
Methodology. **A**, Schematic of dual-source CT scanner gantry showing two separate x-ray sources at 90 degree offsets with example photon energies density distributions demonstrating minimal overlap between the two (140 kVp spectrum has low-energy photons attenuated by a 0.4 mm tin filter). Points A–D represent examples of imaged objects with distinct compositions. Point A is 100% gas and is reliably interpreted as –1,000 Hounsfield units (HU) at both energy levels. Point B, however, is composed of three different materials but is interpreted as 58 HU at 140 kVp, the same as a voxel comprising 100% soft tissue (point D). When points B and D are imaged at 80 kVp, they have different CT densities (78 and 62 HU), thus the materials can be differentiated. A similar argument exists for point C. **B**, In general, after plotting the CT densities of all voxels in an image (here one of the volume scans used for this paper), various distributions can be seen. Point E—100% gas; F—100% soft tissue; G—100% iodinated blood; H—CT scanner table; I—bone. All voxels containing a mix of purely gas and soft tissue fall along the identity line; however, if iodine is added, they are displaced from this line, thus allowing the composition of the voxel to be identified. **C**, Normalization of dynamic dual-energy CT (DECT) gas and iodinated blood volumes to lung tissue mass. Individual frames were scaled up to the size of the whole lung using a scale factor defined as the ratio of the entire thorax to the slice and then divided by the mass of soft tissue in the whole lung. Whole lung gas volumes were used to calculate fractional atelectatic mass in expiration, cyclical recruitment/derecruitment (R/D), and overdistended volume.

We developed a three-material differentiation algorithm for DECT images, validated it in vitro and in vivo, and used it to study a collapse-prone lung injury pig model. We hypothesized that ventilatory conditions associated with significant atelectasis and minimal tidal recruitment/derecruitment (R/D), as results from low PEEP in our model, would demonstrate worsening of gas (V) and blood volume (Q) matching during inspiration.

## MATERIALS AND METHODS

Animal experiments received ethics committee approval (Uppsala Regional Animal Research Ethics Committee ref. C98/16) and conformed with the Animal Research: Reporting of In Vivo Experiments (ARRIVE) (17) guidelines. For full experimental details, see **Supplementary Methods** (Supplemental Digital Content 1, http://links.lww.com/CCM/F147).

### Experimental Protocol

Seven domestic pigs (28.7 kg [2.1 kg]; mean [sd]) were mechanically ventilated under general anesthesia and a lung injury model induced by saline-lavage surfactant depletion. Animals were ventilated supine in a protocolized order covering PEEP steps from 5 to 20 cm H_2_O, in 5 cm H_2_O increments, and in reverse to 0 cm H_2_O (from here on termed “ventilatory conditions”). Both limbs of the incremental/decremental PEEP protocol were studied in order to detect any hysteresis in the results, and the protocol inverted in two animals to reduce bias related to time from injury. Respiratory rate was 10 min^–1^, tidal volume (Vt) 10 mL/kg and inspiratory:expiratory ratio 1:2. Single juxtadiaphragmatic slice dDECT images of two complete respiratory cycles were obtained at 1-second intervals in each ventilatory condition. Images were segmented into three gravitational regions of equal height, and the DECT algorithm (Supplemental Methods, Supplemental Digital Content 1, http://links.lww.com/CCM/F147) applied to determine the mean volume fractions of gas, iodinated blood, and soft tissue within each region.

### Normalized Gas and Blood Volumes

When the lung is inflated, it expands in three dimensions, but only two of these dimensions are included within a single CT slice. Gas and blood volumes within each region were therefore normalized to lung tissue mass. The ratio between the thoracic cavity and slice volumes was used as a scaling factor to approximate whole lung gas and blood values. Whole lung-equivalent values were then divided by the per-animal mean lung tissue mass within each region measured using volume CT scans (**Fig. 1*C***).

### Fractional Atelectatic Mass

Whole lung volume DECT scans were obtained during end-expiratory apneas in each ventilatory condition. Atelectatic subregions were defined as regions with gas volume fraction less than or equal to 0.1 (equivalent to regions ≥ –100 HU on single-energy noncontrast scans [18]). Volume and mean tissue density (1–gas density) were used to calculate the masses of atelectatic subregions and the whole lung. The ratio of these two masses was termed fractional atelectatic mass in expiration (FAM_exp_) (19) and used to categorize the ventilatory conditions (FAM_exp_ < 20%, 20–40%, and ≥ 40%).

### Statistical Analyses

Comparisons between two groups were performed using *t* test or Wilcoxon signed-rank test and those between three groups using Tukey test. Correlation between independent and dependent variables was assessed with linear regression analysis following assessment of individual variables for normality and heteroscedasticity. Correlations involving FAM_exp_ were examined using Spearman rank correlation coefficient due to non-normality in FAM_exp_.

## RESULTS

The DECT algorithm was validated in vitro and in vivo (**Supplementary Figs. 1–5**, Supplemental Digital Content 1, http://links.lww.com/CCM/F147). Briefly, the algorithm accurately predicted blood iodine concentrations in vitro (*r*^2^ = 0.998; *p* < 0.0001; *n* = 4) and provided reasonable agreement in lung volume changes compared with spirometry in vivo (*r*^2^ = 0.92; mean error, –33 mL [95% CI, –38 to –28 mL]; *n* = 8), without being affected by cumulative iodine doses up to 9.2 g/kg or end-expiratory lung volumes between 166 and 1,673 mL. Single slice mean tissue density was correlated with, but consistently less than, equivalent whole lung densities (*r*^2^ = 0.97; relative decrease 14.3% [13.4–15.2%]; *n* = 7); this difference was consistent between inspiration and expiration (*n* = 5). The imaged slice moved caudally during inspiration by a mean of 3.21 mm (2.76–3.66 mm), and it never moved by a distance greater than the adjacent slice moving into the CT image.

### Baseline Characteristics and Cardiorespiratory Variables

Mean pulmonary artery pressure always exceeded mean airway pressure (mean difference 20.3 mm Hg [7.2 mm Hg]). Cardiac output was 3.41 L/min (0.40 L/min), similar to the value of 3 L/min chosen to determine iodine contrast infusion rate. PEEP was positively correlated with peak airway pressure (*r*^2^ = 0.73) and negatively correlated with FAM_exp_ (*ρ* = –0.90). Data points from each of the seven animals were included within each FAM_exp_ group. **Supplementary Tables 1–3** (Supplemental Digital Content 1, http://links.lww.com/CCM/F147) present details of cardiorespiratory variables grouped by animal, PEEP, and FAM_exp_. The hysteresis between the limbs of the PEEP trial is presented in **Supplementary Figure 6** (Supplemental Digital Content 1, http://links.lww.com/CCM/F147), and lung compliance and driving pressures throughout the PEEP trial presented in **Supplementary Figure 7** (Supplemental Digital Content 1, http://links.lww.com/CCM/F147).

### Effect of Inspiration Upon Gas and Iodinated Blood Volume Fractions

**Figure 2.**
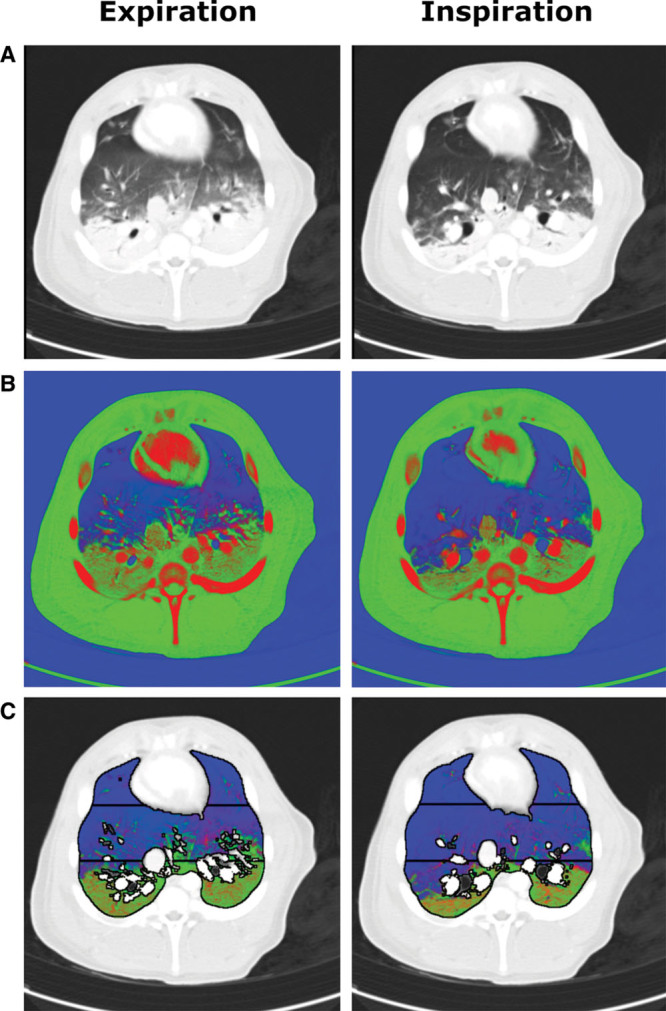
Example source and post-processed images of a single juxtadiaphragmatic slice at positive end-expiratory pressure 5 cm H_2_O of pig’s thorax during iodine infusion using the dual-energy CT (DECT) algorithm. **A**, Composite source images representing a 30:70 merge of 80 kVp and 140 kVp images displayed using standard CT lung windows. **B**, Results of the DECT three-material differentiation algorithm for gas (*blue*), soft tissue (*green*), and iodinated blood (*red*) volume fractions. **C**, The DECT images following segmentation to include only lung parenchyma with the three gravitational regions of interest displayed. Typical expiration and inspiration images are shown in each case. A gravitational effect was seen within the slice with soft tissue and iodinated blood concentrated toward the dependent regions, with a reduction in volume fractions of these materials in inspiration.

**Figure 3.**
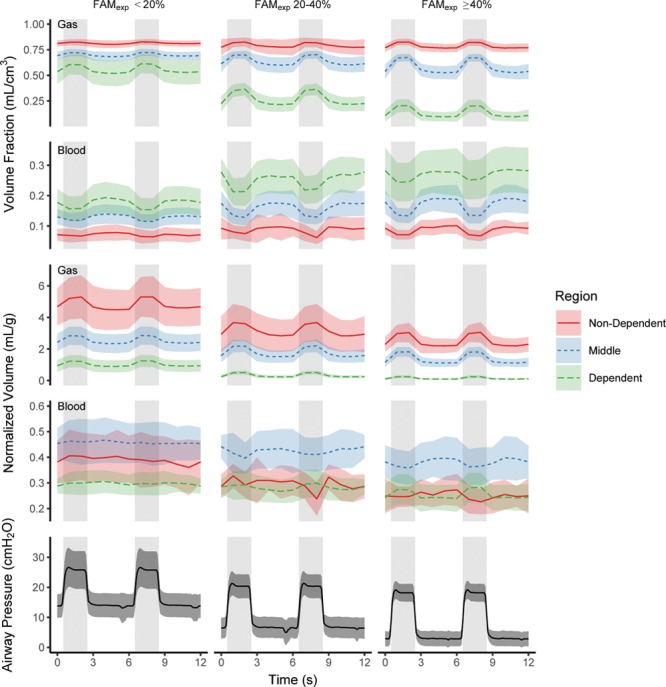
Effects of inspiration on the volume fractions and normalized volumes of gas and iodinated blood within the juxtadiaphragmatic slice over the course of two respiratory cycles. Results are presented for the three different gravitational regions of the studied slice and grouped by fractional atelectatic mass of the lung in expiration (FAM_exp_). Airway pressure traces are provided for comparison, and *gray* background denotes inspiration. In all regions and all FAM_exp_ groups gas volume fraction and normalized gas volume increased (*p* ≤ 0.01) and blood volume fraction decreased (*p* < 0.005) during inspiration. The effects of inspiration on normalized blood volume were most pronounced in the FAM_exp_ greater than or equal to 40% group, with normalized blood volume decreasing in the middle and nondependent regions and increasing in the dependent region. Points represent mean and sd.

A gravitational effect on the distributions of gas, blood, and soft tissue volume fractions within the slice was seen (**Figs. 2** and **3**). Iodinated blood and soft tissue predominated in the dependent regions and gas in the nondependent regions, this effect being more pronounced in the higher FAM_exp_ groups (Fig. 3). During inspiration, gas volume fraction increased in all FAM_exp_ groups and gravitational regions (all *p* ≤ 0.01), and blood volume fraction decreased (all *p* < 0.005). The effect of time into each individual scan sequence on iodinated blood volume fraction was minimal (increase of 0.0007 mL/cm^3^/s [0.4%/s]; *p* = 0.01; *r*^2^ = 0.006).

### Effect of FAM_exp_ Upon Expiratory Normalized Gas and Blood Volumes

**Figure 4.**
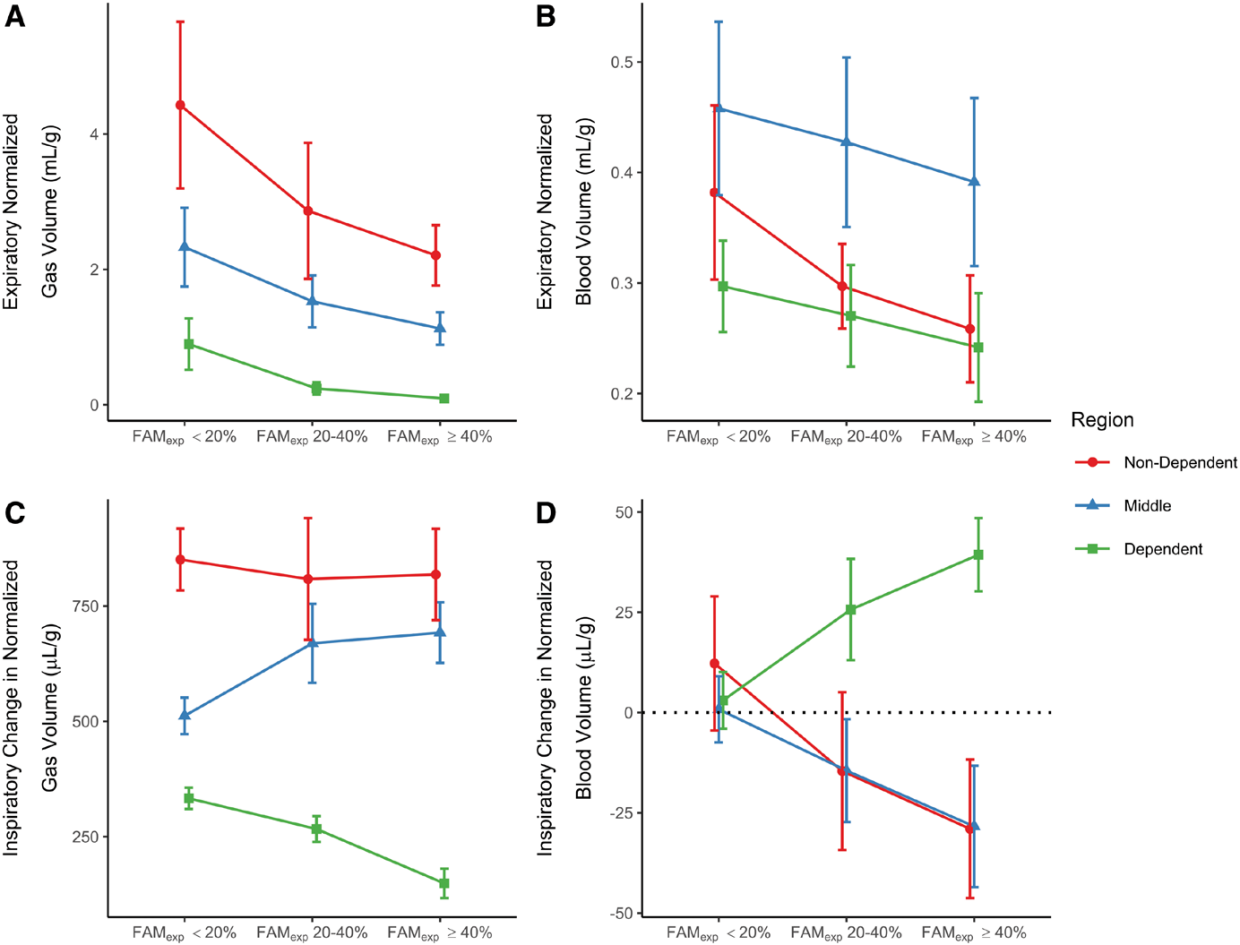
Absolute and relative changes in expiratory normalized gas (V_N_) and blood volumes (QN). V_N_ (**A**) and Q_N_ (**B**) within each region, and fractional expiratory mass of the lung in expiration (FAM_exp_) grouping. **C**, Effects of an inspiratory breath upon V_N_. In the higher FAM_exp_ groups there is relatively less ventilation occurring in the dependent regions. **D**, Effects of an inspiratory breath upon Q_N_. Minimal change was seen in normalized blood volume in the FAM_exp_ less than 20% group, however, in the other conditions the normalized blood volume in the dependent region increased and those in the others decreased with inspiration. Points represent mean and either sd (**A** and **B**) or 95% CI of change (**C** and **D**).

Expiratory normalized gas volume (V_N_) was greatest in the nondependent region and least in the dependent region in all FAM_exp_ groups (**Fig. 4*A***; all *p* < 0.0006), and higher within all regions in the FAM_exp_ less than 20% group compared with the other two FAM_exp_ groups (all *p* < 0.0008). Expiratory normalized blood volume (Q_N_) was greatest in the middle region in all FAM_exp_ groups (**Fig. 4*B***; all *p* < 0.0002). Within-region, expiratory Q_N_ was always highest in the FAM_exp_ less than 20% group compared with the greater than or equal to 40% group (all *p* < 0.027). Similar effects were seen when data were grouped by PEEP, with V_N_ and Q_N_ distributed within the low PEEP groups similarly to high FAM_exp_ groups (Supplementary Fig. 6, Supplemental Digital Content 1, http://links.lww.com/CCM/F147).

### Effects of Inspiration Upon V_N_ and Q_N_

Inspiration was associated with an increase in V_N_ (ΔV_N_) within all regions in all FAM_exp_ groups (Figs. 3 and 4*C*; all *p* < 0.0003). The increase was greater in the nondependent region compared with the dependent region in all cases (*p* < 0.0001; Fig. 4*C*). The FAM_exp_ greater than or equal to 40% group demonstrated the greatest variation in ΔV_N_ between the nondependent and dependent regions (818 µL/g [729–908 µL/g] vs 149 µL/g [120–178 µL/g], respectively; *p* < 0.0001).

Total Q_N_ within the slice was not affected by inspiration (ΔQ_N_ in FAM_exp_ < 20% group: 5 µL/g [–1 to 12 µL/g]; FAM_exp_ 20–40%: –1 µL/g [–9 to 7 µL/g]; FAM_exp_ ≥ 40%: –6 µL/g [–15 to 3 µL/g]). In the FAM_exp_ greater than or equal to 40% group, Q_N_ decreased in the nondependent (29 µL/g [12–46 µL/g]; *p* = 0.02) and middle (28 µL/g [13–44 µL/g]; *p* = 0.01) regions, but increased in the dependent region (39 µL/g [30–48 µL/g]; *p* < 0.001). In the FAM_exp_ 20–40% group, ΔQ_N_ in the dependent region was 26 µL/g (13–38 µL/g) (*p* = 0.01). There was no inspiration-related change in Q_N_ in any region in the FAM_exp_ less than 20% group (*p* = 0.5, 0.8, and 0.8; **Fig. 4*D***).

**Figure 5.**
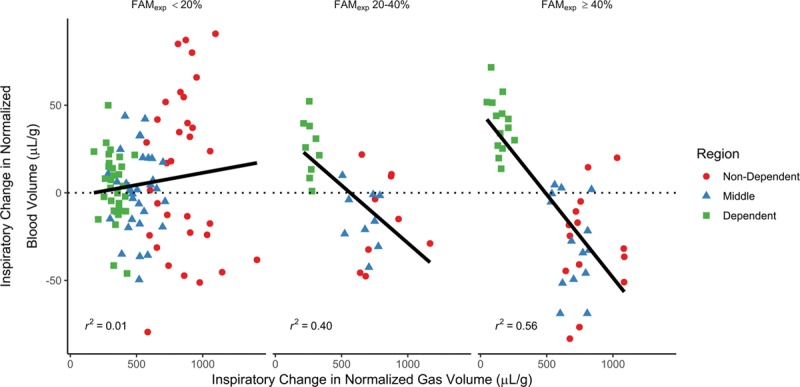
Relationship between the inspiratory change in normalized gas and blood volumes dependent upon fraction of the mass of the entire lung that was atelectatic in expiration (FAM_exp_). For FAM_exp_ less than 20% minimal relationship was seen; however, within the other two groups there was a clear negative relationship: those regions with the least ventilation received an increase in blood volume and those with the most ventilation a decrease, suggestive of an inspiration-related redistribution that worsened ventilation-perfusion matching.

A negative relationship between regional ΔV_N_ and ΔQ_N_ was observed in the FAM_exp_ greater than or equal to 40% (**Fig. 5**; *r*^2^ = 0.56) and FAM_exp_ 20–40% (*r*^2^ = 0.40) groups. FAM_exp_ and ΔQ_N_ in the dependent region were positively correlated (*ρ* = 0.79).

### Effects Upon Pao_2_/Fio_2_ Ratio

**Figure 6.**
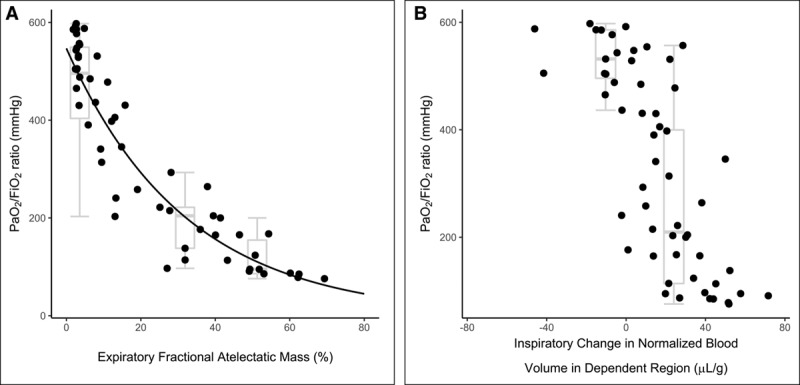
Pao_2_/Fio_2_ (P/F) values associated with atelectasis and blood volume redistribution. Effect of atelectasis (**A**) and intra-tidal normalized blood volume redistribution toward the dependent region (**B**) upon P/F ratio. P/F ratio was negatively correlated with both measures in a nonlinear fashion (Spearman *ρ* = –0.93 and –0.77, respectively) and the log-transform of P/F ratio was linearly related to atelectasis (*r*^2^ = 0.87). *Box*-and-*whisker* plots represent median, interquartile range and range for the three different fractional atelectatic mass in expiration (FAM_exp_) groups studied (**A**) and between those conditions that demonstrated either an inspiration-related reduction or increase in blood volume in the dependent region (**B**).

Pao_2_/Fio_2_ (P/F) ratio was negatively correlated with both FAM_exp_ (*p* < 0.0001; *ρ* = –0.93) and ΔQ_N_ in the dependent region (*p* < 0.0001; *ρ* = –0.77). The relationships were nonlinear in both cases (**Fig. 6**). Following log-transformation of P/F ratio values, a linear relationship with FAM_exp_ was demonstrated (*r*^2^ = 0.87).

## DISCUSSION

We found that inspiratory mechanical breaths at PEEP levels associated with clinically significant atelectasis and minimal tidal R/D cause a redistribution of pulmonary parenchymal blood volume toward poorly ventilated regions in experimental collapse-prone lung injury. This phenomenon would increase shunt fraction beyond what would be expected from atelectasis alone and may represent a significant causal component of the hypoxemia observed with low PEEP ventilation in ARDS (2).

### Methodology Developed for This Study

We developed a DECT three-material differentiation algorithm to quantify gas and blood volume fractions at the voxel level. The iodine infusion protocol caused near-constant opacification of the entirety of the pulmonary vascular tree over the time course of the scan. The validity of the three-material differentiation algorithm was confirmed in vivo and in vitro (**Supplementary Materials**, Supplemental Digital Content 1, http://links.lww.com/CCM/F147), and the normalization procedure to convert volume fractions of gas or blood to volumes per unit mass of lung tissue produced V_N_ values with a typical gravitational gradient (Fig. 4*A*) and hysteresis (Supplementary Fig. 6, Supplemental Digital Content 1, http://links.lww.com/CCM/F147). Following normalization to tissue mass, the middle region of the lung had the highest blood volume (Fig. 4*B*). This agrees with MRI results in human volunteers, where perfusion per unit tissue mass was greatest in the middle gravitational region (6 mL/g/min) compared with the dependent and nondependent regions (4–5 mL/g/min) (20). Overall, these findings demonstrate the usefulness of our technique and the dependability of the results.

### Intra-Tidal Blood Volume Redistribution

In conditions with large volume atelectasis (≥ 40% of lung mass), we demonstrated minimal tidal R/D (< 7% of lung mass). The majority of recruitment takes place over 2 seconds from the start of an end-inspiratory apnea; however, around 10% still takes longer than this (21, 22). We calculated tidal R/D based upon volume CT scans during prolonged end-expiratory and end-inspiratory apneas (near-maximal derecruitment and recruitment, respectively). The tidal R/D values reported here may, therefore, overestimate what was occurring during tidal ventilation with an inspiratory time of 2 seconds. We refer to the tidal R/D seen in our study as “minimal” because in the FAM_exp_ greater than or equal to 40% group, it represented around only 1/8 of the total atelectasis caused by PEEP variations (Supplemental Table 3, Supplemental Digital Content 1, http://links.lww.com/CCM/F147). This greater effect of PEEP than Vt in the saline-lavage model is in keeping with previous results (23).

In conditions of large volume of atelectasis with minimal tidal R/D, we demonstrated an inspiration-related reduction in Q_N_ within the nondependent and middle regions associated with a reciprocal increase in the most-dependent region (Fig. 4*D*), in the context of no inspiration-related change in total Q_N_. This suggests a cyclical redistribution of blood volume toward the most-dependent region during inspiration, then restored during expiration. These results contrast those reported in an uninjured rabbit model, where blood volume redistributed from dependent to nondependent regions in inspiration (24). Apart from anatomical differences between models, an explanation for these differences is that, unlike the earlier study, we studied a lung injury model and normalized the results to lung tissue mass.

The dependent region was ventilated least when significant atelectasis was present (Fig. 4*C*), in keeping with results from electrical impedance tomography, where decreasing PEEP (25) or inspiratory time (26) shifts the center of ventilation toward nondependent regions. Inspiratory positive pressures may be delivered only to ventilated alveoli and the inspiration-associated decrease in alveolar vessel transmural pressure and volume only occurs in those regions of the lung that are ventilated, and therefore redistribution of blood to nonventilated regions is likely (13). As the oxygen reservoir within the lungs is highest during inspiration, this redistribution of blood volume would increase shunt fraction. This mechanism could explain why some patients exhibit hypoxemia that is refractory to increases in inspired oxygen concentration and/or inspiratory time.

### Effects of Blood Volume Redistribution on Oxygenation

Increase in CT-measured atelectasis has a negative relationship with oxygenation (27) and with the P/F log-transform (28), as confirmed here (Fig. 6*A*). Furthermore, we demonstrated a negative relationship between the increase in blood volume within the dependent region during inspiration and oxygenation (Fig. 6*B*).

Due to multicollinearity between FAM_exp_ and intra-tidal pulmonary blood volume redistribution, identifying the relative contributions of these two determinants of hypoxemia is challenging. This finding raises an interesting question: is it the presence of atelectasis that causes hypoxemia in ARDS, or is intra-tidal redistribution of blood to atelectatic lung an additional requirement?

### Limitations

We measured aeration and blood volume, surrogates of ventilation and perfusion. Regional ventilation can be derived using our technique by measuring aeration in both inspiration and expiration. Perfusion is more difficult to measure, however, DECT-derived pulmonary blood volume can approximate perfusion (measured by contrast-bolus dynamic CT) with a mean correlation coefficient of 0.7 (29).

We imaged one slice rather than the whole lung due to limitations in current technology. The slice we chose reasonably approximates the lung in terms of atelectatic fractions (30) and density distributions (31) and has been used to quantify atelectatic lung in both the uninjured animal (23, 32) and that with lung injury (23, 33). We demonstrated that while the single slice underestimated lung density, it did so by a consistent amount between inspiration and expiration (**Supplementary Results**, Supplemental Digital Content 1, http://links.lww.com/CCM/F147) such that our final outcome variables (ΔV_N_ and ΔQ_N_) were likely similar to those seen for the whole lung. The minimal inspiration-related caudal displacement of the slice is also reassuring for the validity of these results.

The saline-lavage surfactant-depletion lung injury model demonstrates significant recoverability with both time since injury and application of high PEEP (34, 35). As we used PEEP purely to generate differing amounts of atelectasis in the animals, any recovery was accounted for by the use of FAM_exp_ as a grouping variable, rather than PEEP itself. Additionally, we inverted the PEEP sequence in two animals to minimize any bias induced by recovery purely due to time from injury. The inherent effects of PEEP and inspiratory pressures upon regional blood volume may differ between models, however, and these results should be confirmed in other lung injury models, such as those which demonstrate other aspects of ARDS including regions of nonrecruitable lung.

We investigated a limited number of ventilatory conditions (dorsal recumbency, and fixed respiratory rate and Vt). The respiratory rate could not be increased above 10 min^–1^ due to the current maximum dDECT scanning frequency of 1 Hz. The Vt of 10 mL/kg is reasonable given the greater resting Vt and minute ventilation of the pig compared with humans (36). Dorsal recumbency was chosen as the majority of patients with ARDS are ventilated supine with prone positioning reserved as rescue therapy (37). These results should be confirmed in other ventilatory conditions, particularly Vt variations.

## CONCLUSIONS

We demonstrated a redistribution of pulmonary blood volume away from well-ventilated regions of lung during inspiration in experimental lung injury at PEEP levels associated with significant atelectasis and minimal tidal R/D. This redistribution was associated with a clinically significant reduction in P/F ratio. This intra-tidal pulmonary blood volume redistribution has not previously been demonstrated during mechanical ventilation at clinically-relevant respiratory rates. It may be a putative explanation for the reduced Pao_2_ seen in low PEEP ventilation in ARDS (2), and could potentially explain the large intra-tidal Pao_2_ oscillations seen in experimental lung injury (32, 33, 38–40). Further work examining mechanical ventilatory strategies in ARDS should also examine their effects on pulmonary blood volume distribution, which is also relevant for oxygenation.

## ACKNOWLEDGMENTS

We are grateful to Agneta Roneus, Kerstin Ahlgren, Mariette Anderson, Liselotte Pihl, Maria Swälas, and Monica Segelsjö at Uppsala University Hospital for their expertise and technical assistance; Clive Hahn, Keith Dorrington, Peter Robbins, and Jose Venegas for helpful discussions and Oxford Optronix for technical support. Finally, we are thankful to the unnamed reviewers who have provided highly constructive advice to improve this article.

## Supplementary Material


