# Supplementary material for: Mechanical Ventilation Redistributes Blood to Poorly Ventilated Areas in Experimental Lung Injury*
Source: Crit Care Med. 2020 Feb 13;48(3):e200–8. doi: 10.1097/CCM.0000000000004141 (PMC7017942; doi:10.1097/CCM.0000000000004141)
Supplement: Supplementary file 1 [file ccm-48-e200-s001.pdf]

Mechanical ventilation redistributes blood to poorly ventilated areas in experimental lung injury

John N. Cronin, Douglas C. Crockett, Andrew D. Farmery, Göran Hedenstierna, Anders Larsson, Luigi Camporota, Federico Formenti

Online Data Supplement

1. Supplementary Methods

PEEP trial/experimental protocol

Seven domestic pigs (weight 28.7 (2.1) kg; mean (SD)) were studied at the Hedenstierna Laboratoriet, University of Uppsala, Sweden under general anaesthesia and mechanical ventilation via tracheostomy. Following pre-medication with intramuscular xylazine 2 mg/kg, ketamine 20 mg/kg and midazolam 0.5 mg/kg, an ear vein was cannulated and general anaesthesia induced with intravenous propofol titrated to effect. Muscle relaxation was maintained with continuous infusion of rocuronium and anaesthesia maintained with intravenous infusion of ketamine, fentanyl and midazolam. Adequacy of anaesthesia was confirmed by the absence of cardiovascular signs of sympathetic stimulation. Normovolaemia was maintained with continuous intravenous infusion of Ringer's lactate solution, initially at 20 mL/kg/hr for one hour followed by 10 mL/kg/hr for the duration of the experiment. A surfactant-depletion model of lung injury was achieved using the technique of Lachmann (1) aiming for a PaO_2/FiO_2 ratio (P/F ratio) of 150 mmHg at PEEP 5 cmH_2O and an FiO_2 of 0.7.

Mechanical ventilation was performed using a Servo-I ventilator (Maquet, Rastatt, Germany) with the animals in dorsal recumbency in pressure control mode aiming for a tidal volume of 10 mL/kg, respiratory rate 10 breaths/minute, inspiratory:expiratory (I:E) ratio of 1:2 and inspiratory rise time of 0 s such that each breath consisted of 2 s of inspiration and 4 s of expiration. PEEP was titrated from 5 to 20 cmH_2O and then down to 0 cmH_2O in 5 cmH_2O increments in 5 animals and from 20 cmH_2O to 0 then back up to 15 cmH_2O in 2 animals. Following 5 min of ventilation at each PEEP level, iodine contrast agent (iomeprol; Iomeron 400 mg/mL; Bracco Imaging Scandinavia AB, Göteborg, Sweden) was infused into the superior vena cava at a rate of 300 mg/s starting 16 s prior to CT image acquisition and continuing for the duration of the scan. This protocol was designed to achieve pulmonary artery iodine concentrations of 6 mg/mL assuming a cardiac output of 3 L/min (similar to those seen in this study) and the time delay chosen to ensure that iodine had time to travel through the lung parenchyma, pulmonary veins, left heart and be visible in the descending thoracic aorta. The time between scans was chosen to be at least three times the time constant for the rapid redistribution phase of iomeprol (2) such that there would be minimal intravascular variability in iodine concentration during each scan due to redistribution of contrast administered for a previous scan.

Dual-energy CT scans were then obtained of a single juxtadiaphragmatic slice of the lung at tube voltages of 80 and 140 kVp using a Somatom Definition Flash dual-source CT scanner (Siemens, Erlangen, Germany) at 1 Hz temporal frequency for 18 s such that at least two complete ventilatory cycles were obtained at each PEEP level. Reconstituted voxel size was 0.5x0.5x5 mm.

Continuous ECG, invasive systemic, central venous and pulmonary arterial blood pressures (IntelliVue M8004A, Philips Healthcare, Best, Netherlands), airway flow and pressure (Capnomac Ultima, Datex-Ohmeda, Madison, WI) and continuous pulse contour cardiac output (PiCCO, Pulsion Medical Systems, Munich, Germany) measurements were recorded throughout the PEEP trial and analogue signals interfaced to a PC using a PowerLab analogue-to-digital converter (ADInstruments, Dunedin, New Zealand) and recorded using LabChart version 8 (ADInstruments, Dunedin, New Zealand). Pulmonary artery flotation catheter thermodilution cardiac output measurements and spot arterial blood gas analyses were taken before and after the PEEP trial sequence.

Three-material differentiation algorithm

DECT images were post-processed using a bespoke algorithm that takes as inputs CT images of the same region of lung taken at two energy levels and six ‘DECT coefficients’, which represent the CT attenuation coefficients of three materials of interest at each energy level. For this study, the materials of interest were chosen as gas, soft tissue and iodinated blood. The DECT coefficients for gas were fixed at -1000 Hounsfield Units (HU) for each energy level as per the definition of HU. The coefficients for soft tissue were chosen as the mean attenuation of the liver during each scan series and those for iodinated blood as the mean attenuation of the descending aorta in the last frame of the series. These two structures were chosen because the liver represents a well-perfused soft-tissue organ with a reasonably radiologically homogenous parenchyma similar to the lung with the notable exception that it does not contain gas, and the contents of a large blood vessel can be assumed to contain only blood. These choices of coefficients ensure that any parenchymal and intravascular accumulation of iodine across ventilatory conditions was accounted for in the calculations. The algorithm then attempts to find the optimum values for the fraction of each material that comprise each voxel of the source images with the constraints that each fraction is between 0

and 1 and all three fractions sum to 1. The algorithm used is available under a permissive licence (3).

CT segmentation and analysis

Following three-material differentiation, images were manually segmented in 3D Slicer version 4.8.1 (4) to include lung parenchyma and exclude extra-thoracic contents, the heart, mediastinal contents, inferior vena cava and major airways and bronchi. Each individual image was then classified as recorded either in inspiration or expiration, or marked as intermediate based upon the mean gas volume fraction of the total slice. Specifically, for each ventilatory condition in each animal, the mean gas volume fraction for each 1 Hz frame was linearly scaled to lie between 0% and 100% where 0% represented the mean gas volume fraction of the frame with the least amount of gas and 100% represented that of the frame with the greatest. All frames with a gas volume fraction of ≤30% on this scale were deemed to represent expiration and all with a gas volume fraction ≥70% were identified as inspiratory frames. Images were automatically segmented further into three regions of equal height aligned along the gravity vector and regional volume fractions of gas and iodinated blood calculated.

Calculation of normalized gas and blood volumes

Individual region gas or blood volumes within each slice (V or Q respectively) were calculated based upon the known volume fractions of gas or blood within each region and the volume of the region:

$$V = Gas\ Volume\ Fraction \times Region\ Volume$$

$$Q = Iodinated\ Blood\ Volume\ Fraction \times Region\ Volume$$

A scale factor was calculated for each individual ventilatory condition per animal for both inspiration and expiration based upon the total volume of both the slice in the dynamic series and the entire lung (including gas, parenchyma and blood) taken at breath holds in either inspiration or expiration respectively:

$$Scale\ Factor_{condition} = \frac{Total\ Thoracic\ Volume}{Total\ Slice\ Volume}$$

Whole lung scans taken in expiration at each PEEP setting were also used to determine regional lung tissue mass. The volume was manually segmented to include only the lung itself and exclude extra-thoracic contents and then automatically segmented into three

gravitational regions as described above for a single slice. The mass of each region was calculated for each breath hold by assuming that the water density of the slice was 1 minus the gas volume fraction, and then mean lung mass of each region was calculated. ‘Whole-lung equivalent’ gas and blood volumes were calculated for each region such that they represented the expected volume of gas or blood within the entire lung should the composition of the lung be the same as that of the slice. These were then normalized to the mean tissue mass in that region for that particular animal.

$$V_N = \frac{V \times Scale\ Factor_{condition}}{Whole\ Lung\ Region\ Mass_{animal}}$$

$$Q_N = \frac{Q \times Scale\ Factor_{condition}}{Whole\ Lung\ Region\ Mass_{animal}}$$

Where V_N and Q_N represent normalized gas and iodinated blood volumes within a particular region of the lung respectively.

The entire process is summarised in Fig. 1c.

Measurement of atelectatic mass fractions

Volume DECT scans of the whole lung were obtained during both end-expiratory and end-inspiratory breath holds after acquisition of dynamic images in each ventilatory condition. Each breath hold was 10 s in duration and the scan sequence was started once airway flow fell to zero. Volumes were manually segmented to exclude major intra-pulmonary airways and blood vessels, and extra-thoracic and mediastinal contents. The three-material differentiation algorithm was then applied. Atelectatic subregions were defined as those regions with gas volume fraction ≤ 0.1 (equivalent to regions ≥ -100 Hounsfield Units (HU) on single-energy non-contrast scans as previously described (5)). The mass of this subregion and of the whole lung were calculated based upon region volume and mean tissue density (defined as 1 – gas density). This density is equivalent to the sum of the soft tissue and iodinated blood fractions, assuming each has the density of water. This calculation was chosen, rather than soft tissue alone, to allow comparison with published single-energy CT results where it is impossible to distinguish between soft tissue and blood volume (5). Finally, the fractional atelectatic mass (FAM) was determined as the ratio of the mass within the atelectatic subregion to that of the whole lung and reported in both end-expiration (FAM_{exp}) and end-inspiration (FAM_{insp}). The difference between these two measurements

for each ventilatory condition in each animal was reported as cyclical recruitment/derecruitment (R/D).

Validation experiments

Eleven 10 mL plastic syringes were prepared containing heparinized pig's blood mixed with iodine contrast agent at concentrations between 0 and 10 mg/mL in 1 mg/mL increments and DECT images obtained as previously described. The CT attenuation coefficients of non-iodinated (0 mg/mL) and iodinated (10 mg/mL) blood were measured as the mean of 3 mm radius x 30 mm depth (six 5 mm slices) cylinders concentric with the relevant syringe and these values used as the DECT coefficients for non-iodinated and iodinated blood respectively. Mean voxel iodinated blood fraction for a similar sized cylindrical sample of each syringe content was then obtained and multiplied by 10 mg/mL to give a final DECT-derived iodine concentration.

For *in vivo* validation of gas volumes a whole lung DECT scan (voxel size 0.5x0.5x5 mm) of pig thorax was obtained during breath holds at end-inspiration, followed by a release of lung volume to end-expiration, without any intervening breaths. Images were segmented to exclude extra-thoracic and mediastinal contents, and the inferior vena cava and included trachea up to the level of the tip of the tracheostomy tube. DECT coefficients for iodinated blood were taken as the mean CT densities of the mid-thoracic descending aorta at each energy level and soft tissue as the mean CT densities of the liver parenchyma. Mean gas volume fraction was multiplied by total segmented volume to give a gas volume and the difference between inspiratory and expiratory volumes taken as the DECT-derived tidal volume. Spirometry tidal volume was measured at the airway and converted to BTPS conditions prior to comparison with DECT-derived volumes. PEEP was varied between 0 and 20 cmH_2O and approximately 1 minute prior to each scan a dose of iodine contrast was administered to ensure the algorithm gave consistent results for gas volume fraction over a wide range of cumulative iodine doses.

Validation experiments were analysed using simple linear regression and the method of Bland and Altman (6). *In vivo* gas volume experiments were also analyzed using a modification of the Bland-Altman technique where cumulative iodine dose and end-expiratory lung volume were also represented along the x-axis to exclude any non-constant

bias due to these variables. For five animals tidal volume was fixed at 10 mL/kg and PEEP varied between 0 and 20 cmH_2O, and tidal volume was also varied for another three animals.

Single-slice vs whole lung analysis

We sought to demonstrate that the single juxtadiaphragmatic slice chosen was representative of the whole lung in injured animals in both expiration and inspiration. The mean densities of the single slice and whole lung in the seven animals in the main experiment were calculated as the sum of the soft tissue and iodinated blood densities. This is equivalent to 1 minus the air density i.e. similar to the calculation of mean lung density in a two-compartment single-energy CT experiment (5). For each ventilatory condition in each animal mean densities of each slice during expiration and each end-expiratory whole lung volume scan were calculated (n=7) and in five of these animals equivalent volumes in inspiration/end-inspiration were also measured.

In order to assess the cranio-caudal displacement of a single slice of the lung between expiration and inspiration, single-energy volume CT scans were obtained during end-expiration and end-inspiration breath holds in 4 animals with voxel sizes of 0.5x0.5x0.6 mm. The expiration images were then registered onto the inspiration images using NiftyReg software (7), which uses the free-form deformation technique to generate a mesh of B-spline transformations by minimizing a cost function defined as the difference between the moving (expiration) and fixed (inspiration) volumes. It has previously been used to assess regional strain in the lung (8, 9). Following manual inspection of the accuracy of the registration, a 5 mm thick slice of lung at the level of the juxtadiaphragmatic slice used in the dynamic images was segmented in the expiration volume and the mean cranio-caudal displacement in each of 12 antero-posterior regions calculated from the transformation mesh. This experiment was performed at five separate PEEP settings (0, 5, 10, 15 and 20 cmH_2O) in each animal with tidal volumes of 10 mL/kg and the results presented as the cranio-caudal displacement in each region grouped by PEEP setting.

Statistical analyses

Comparisons of gas and blood volume fractions and V_N and Q_N between inspiration and expiration within each region and each PEEP level and additionally between those volumes on the incremental and decremental limbs of the PEEP trial were performed using *t*-test or

Wilcoxon's signed rank test (dependent on normality of variables) with alpha adjustment for multiple comparisons using the method of Holm (10) with the cut-off for statistical significance set at 0.05 with pairing based upon individual animals as appropriate. Incremental vs decremental limb analyses were performed for PEEP levels 5, 10 and 15 cmH_2O only; 0 and 20 cmH_2O were considered to be the end points of the hysteresis loop and thus members of both limbs. Differences between atelectatic mass between incremental and decremental limbs was performed using Wilcoxon's signed-rank test due to non-normality of fractional atelectatic mass. DECT frames that were intermediate between being in inspiration and expiration were excluded from analyses. Linear regression analysis was employed to detect the impact of time upon iodinated blood volume fraction, PEEP upon absolute V_N and inspiratory change in V_N on inspiratory change in Q_N. The effect of PEEP and incremental/decremental limb upon absolute Q_N was examined using two-way analysis of variance with Tukey's honest significant differences post-hoc analysis. Correlations with FAM_{exp} and P/F ratio were performed using Spearman's rank correlation coefficient. All analyses were performed in R version 3.5.0 (11).

2. Supplementary Results

In vitro validation of iodinated blood concentrations

The algorithm accurately predicted iodinated blood concentrations *in vitro* between known concentrations of 0 to 10 mg/mL iodine (Fig. E1a; gradient of regression line 0.96 [95% CI 0.94-0.99]; r^2=0.998; P<0.0001; n=4 per point). Minimal bias was seen throughout all values of known concentration with a slight reduction in predicted values at the upper end of the scale (Fig. E1b; mean difference 0.19 mg/mL [0.07-0.32 mg/mL], 95% limits of agreement -0.16 mg/mL [-0.37-0.33 mg/mL] to 0.55 mg/mL [0.05-0.76 mg/mL]). The overall bias was equivalent to the algorithm overestimating iodinated blood concentrations by 6% in the middle of the range. Gas comprised 0.8% (0.4%) of all syringes.

In vivo validation of gas volumes

Paired spirometry and DECT tidal volumes were collected for 8 animals at a variety of PEEP settings (0–20 cmH_2O), end-expiratory lung volumes (166–1673 mL), mean lung densities (0.27–0.83 g/cm^3) and tidal volumes (4.8–16.2 mL/kg). One animal died during the experiment due to cardiovascular collapse. The two measures were closely correlated (r^2=0.92; P<0.0001; Supplementary Figure 2). Mean error between DECT and spirometry

measurements of tidal volume was -33 mL [-38 to -28 mL] with 95% limits of agreement -76 mL [-85 to -67 mL] and 9 mL [0 to 18 mL]. This bias was consistent over a wide range of tidal volumes (Supplementary Figure 3a), cumulative iodine doses (0.7 – 9.2 g/kg; Supplementary Figure 3b) and end-expiratory lung volumes (Supplementary Figure 3c).

Applicability of single juxtadiaphragmatic slice measurements to whole lung

Mean slice density (calculated as summation of iodinated blood and soft tissue densities) of a single juxtadiaphragmatic slice in expiration and inspiration scanned using DECT during ventilation closely correlated the mean density of the whole lung scanned during end-expiratory and end-inspiratory breath holds (r^2=0.97; P<0.0001; Supplementary Figure 4a). Overall, mean slice density was 14.3% [13.4 to 15.2%] less than mean lung density with 95% limits of agreement between 5.8% [4.2 to 7.3%] and 22.9% [21.4 to 24.5%]. This relative bias was constant over the range of mean densities sampled and between inspiratory and expiratory measures at each ventilatory setting (Supplementary Figure 4b).

Mean displacement of the region of lung contained within the juxtadiaphragmatic slice between end-expiratory and end-inspiratory breath holds was 3.21 mm [2.76-3.66 mm] in the caudal direction. The slice of lung imaged in expiration only moved completely out of the field of view of the CT scanner in some animals in inspiration within the middle region of the lung at PEEP levels of 5, 10 and 15 cmH_2O and in the dependent region at PEEP 10 and 15 cmH_2O and each case the movement was consistent with the adjacent slice moving into the field of view (Supplementary Figure 5).

3. Supplementary Table 1. Cardiorespiratory parameters

Parameter		Animal							Mean
		1	2	3	4	5	6	7	
Weight (kg)		31	32	30	27	27	27	27	28.7 (2.1)
Lavage volume (L)		1	6	3	5	9	5	3	4.6 (2.6)
P/F ratio (mmHg)		110	151	174	110	150	111	101	130 (28)
$EELV_{5\ cmH_2O}$ (mL)		365	464	414	383	388	580	410	444 (70)
Lung mass (g)		632 (82)	713 (39)	629 (16)	577 (30)	662 (36)	590 (22)	645 (11)	636 (45)
$FAM_{5\ cmH_2O}$ (%)		39	46	41	60	51	32	53	46 (10)
ABP (mmHg)	Sys	94 (8)	93 (6)	79 (9)	82 (7)	84 (8)	74 (9)	75 (5)	83 (8)
	Dia	88 (10)	84 (6)	73 (10)	77 (7)	79 (9)	68 (9)	70 (6)	77 (7)
PAP (mmHg)	Sys	29 (2)	32 (4)	31 (3)	35 (4)	38 (3)	38 (5)	33 (3)	34 (4)
	Dia	24 (2)	27 (4)	27 (4)	31 (4)	33 (2)	33 (3)	29 (4)	29 (3)
CVP (mmHg)		10 (2)	12 (2)	13 (3)	14 (3)	10 (1)	9 (1)	10 (1)	11 (2)
HR (bpm)		106 (4)	67 (4)	86 (10)	77 (7)	88 (5)	91 (4)	88 (1)	86 (12)
CO (L/min)		4.13	3.00	3.60	3.22	2.98	3.41	3.52	3.41 (0.40)

Definition of abbreviations: P/F ratio = PaO_2/FiO_2 ratio sampled at an FiO_2 of 0.7 and PEEP 5 cmH_2O; $EELV_{5\ cmH_2O}$ = end-expiratory lung volume at PEEP 5 cmH_2O measured by CT including dead space to the level of the tip of the tracheostomy tube; $FAM_{5\ cmH_2O}$ = fraction of the mass of the entire lung that was atelectatic in expiration (gas volume fraction ≤0.1 mL/cm^3) at PEEP 5 cmH_2O; ABP = systemic arterial blood pressure; PAP = pulmonary arterial pressure; CVP = central venous pressure; HR = heart rate; CO = cardiac output measured by pulmonary artery flotation catheter thermodilution; Sys = systolic; Dia = diastolic. Data are presented as mean (SD) where appropriate (n=8 for individual animal statistics).

4. Supplementary Table 2. Spirometry and atelectasis measurements throughout the PEEP trial

Limb	PEEP (cmH_2O)	Pmean (cmH_2O)	Ppeak (cmH_2O)	VTe (mL)	VTe (mL/kg)	Cstat (mL/cmH_2O)	FAM_{exp} (%)
Incremental	5	10.3 (0.7)	20.1 (2.1)	265 (28)	9.31 (1.21)	17.9 (3.5)	30.7 (13.1)
	10	14.9 (0.7)	24.2 (2.0)	272 (28)	9.52 (1.03)	19.5 (3.1)	11.4 (9.3)
	15	19.5 (0.5)	28.1 (1.5)	279 (41)	9.71 (0.79)	21.9 (3.5)	1.0 (0.8)
	20	25.0 (0.9)	34.7 (2.6)	265 (25)	9.26 (0.83)	18.6 (4.0)	0.6 (0.4)
Decremental	15	17.9 (0.4) *	23.3 (1.0) *	258 (31)	9.01 (0.88)	31.5 (6.2) *	0.5 (0.2)
	10	12.9 (0.3) *	18.3 (0.8) *	251 (35)	8.75 (0.92)	30.5 (5.5) *	2.7 (3.8) *
	5	9.2 (0.2) *	16.9 (0.8) *	260 (25)	8.98 (0.65)	22.0 (3.3) *	26.2 (11.2) *
	0	5.8 (0.6)	16.4 (1.9)	258 (25)	8.90 (0.97)	16.2 (3.1)	41.2 (10.7)

Definition of abbreviations: PEEP = positive end-expiratory pressure; Pmean = mean airway pressure; Ppeak = peak airway pressure; VTe = expiratory tidal volume; Cstat = static compliance; FAM_{exp} = fractional of the total mass of the lung that was atelectatic in expiration (gas volume fraction ≤0.1 mL/cm^3). Data are presented as mean (SD). *n*=7.
*P < 0.05 versus incremental limb.

5. Supplementary Table 3. Characteristics of fractional atelectatic mass groupings

Parameter	FAM_{exp} < 20%	FAM_{exp} 20-40%	$FAM_{exp} \geq 40\%$
Number of ventilatory conditions	31	9	14
PEEP (cmH_2O)	14.2 (4.1)	6.7 (3.5)	2.9 (2.6)
Cstat (mL/cmH_2O)	25.0 (7.2)	19.6 (4.5)	17.8 (3.5)
FAM_{exp} (%)	6.5 (4.9)	31.2 (5.1)	52.4 (8.6)
FAM_{insp} (%)	6.0 (3.9)	27.0 (4.0)	46.8 (8.6)
FOM_{exp} (%)	0.79 (0.64)	0.38 (0.47)	0.16 (0.13)
FOM_{insp} (%)	1.70 (0.86)	1.52 (0.90)	0.95 (0.59)
Cyclical R/D (%)	0.7 (1.5)	5.2 (0.8)	6.4 (1.6)

Definition of abbreviations: FAM = fractional atelectatic mass, i.e. percentage of the mass of the whole lung that is atelectatic; FOM = fractional overdistended mass; PEEP = positive end-expiratory pressure; Cstat = static compliance; FAM_{exp} = FAM during end-expiratory breath hold; FAM_{insp} = FAM during end-inspiratory breath hold; FOM_{exp} = FOM during end-exppiratory breath-hold; FOM_{insp} = FOM during end-inspiratory breath hold; Cyclical R/D = cyclical recruitment/derecruitment measured as the difference between FAM_{insp} and FAM_{exp}. Data are presented as mean (SD).

6. Supplementary Figure 1

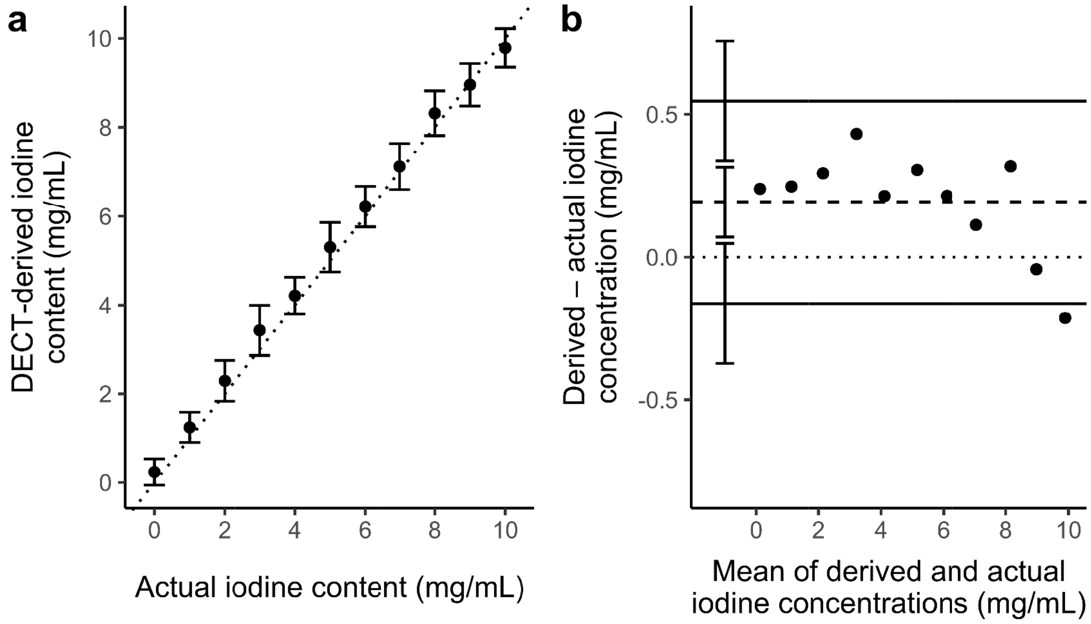

Supplementary Figure 1. Results of DECT algorithm validation *in vitro*. a) Correlation between 11 known iodine concentrations in heparinized pig's blood in syringes and concentration derived by DECT algorithm. Points represent mean of 4 measurements and standard deviation. Dotted line is the identity line. b) Bland-Altman analysis of points from a). Dotted horizontal line represents no difference, dashed line is mean difference, solid lines 95% limits of agreement and error bars 95% confidence intervals.

7. Supplementary Figure 2

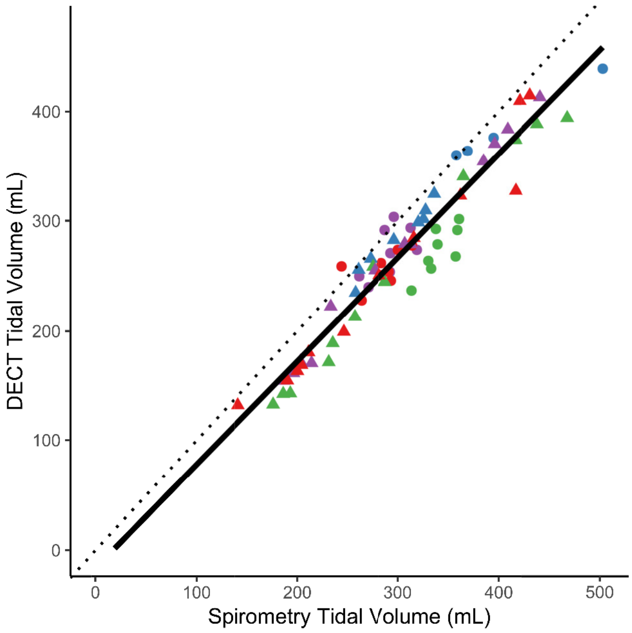

Supplementary Figure 2. Tidal volumes measured by DECT closely correlated those measured by airway spirometry over a range of tidal volumes between approximately 5 and 16 mL/kg (r^2=0.92). Different colours and symbols represent different animals (n=8). Solid line is the regression line and dotted line the identity line.

8. Supplementary Figure 3

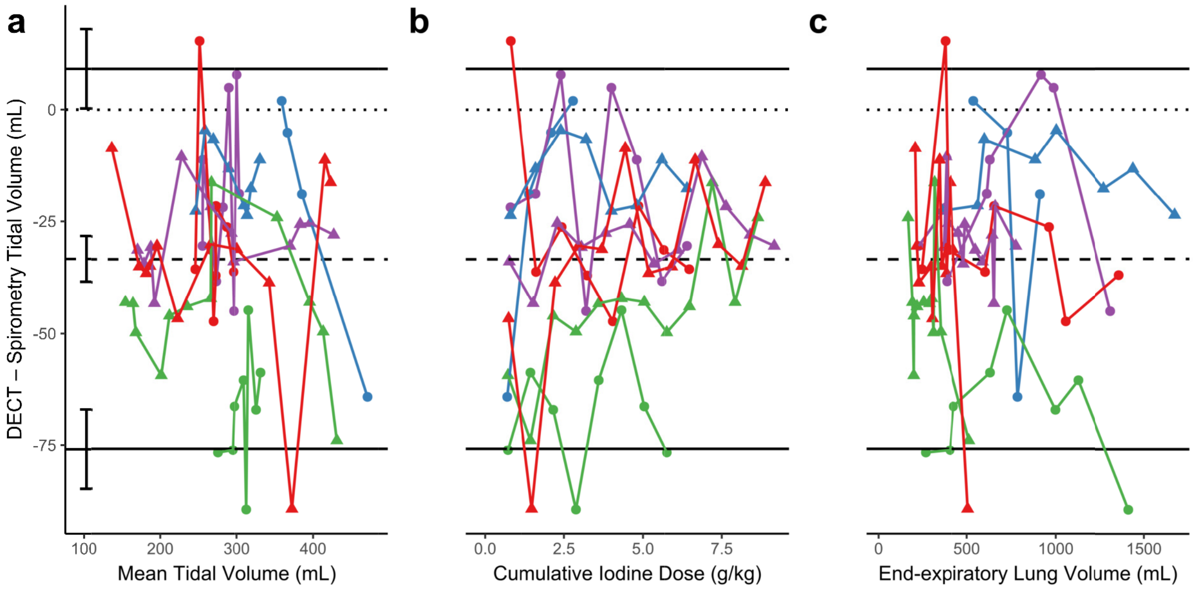

Supplementary Figure 3. Mean bias between tidal volumes measured by DECT and airway spirometry was -33 mL and this was consistent over a range of tidal volumes (a), cumulative iodine doses (b) and end-expiratory lung volumes as measured by DECT (c). Different colours and symbols represent different animals (n=8) with the points from each animal linked by coloured lines. Dotted horizontal line represents no difference, dashed line is mean difference, solid lines 95% limits of agreement and error bars 95% confidence intervals.

9. Supplementary Figure 4

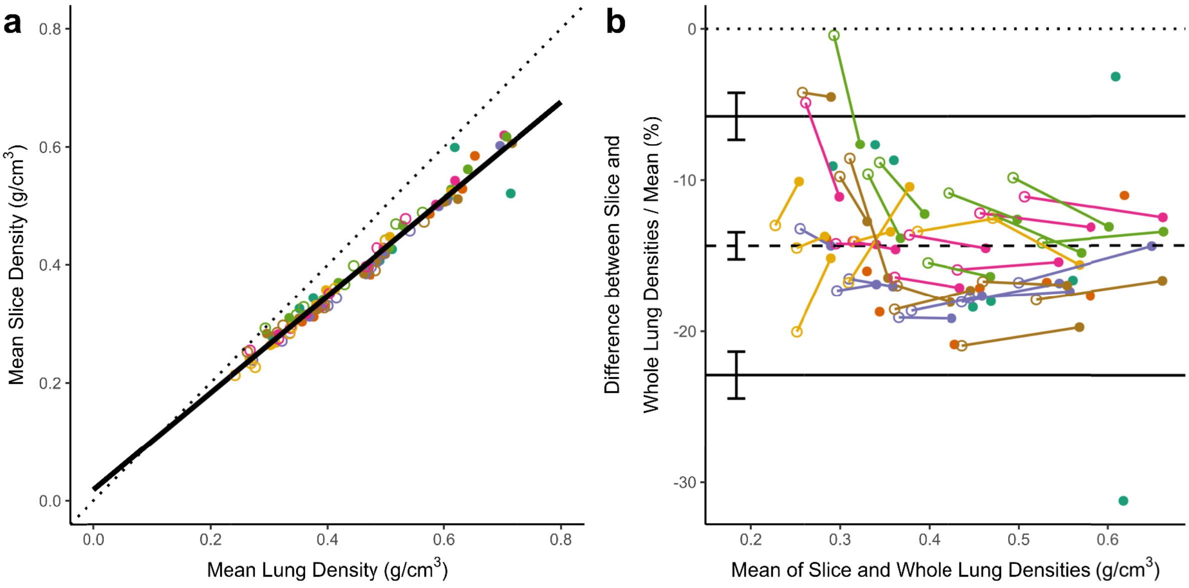

Supplementary Figure 4. a) Mean density of a single 5mm juxtadiaphragmatic slice of lung parenchyma and that of the whole lung were closely correlated (r^2=0.97). Different colours represent different animals, filled circles represent measurements taken during either expiration (for single slice) or during an end-expiratory breath hold (for whole lung) and empty circles those taken during inspiration/end-inspiratory breath holds. Solid line is the regression line and dotted line the identity line. n=7 for expiration and 5 for inspiration. b) Modified Bland-Altman analysis of the points in a) demonstrating constant relative bias of -14% between mean slice and whole lung densities. Colours and symbols as in a) with lines joining expiratory and inspiratory values at the same ventilator settings. Dotted horizontal line represents no difference, dashed line is mean difference, solid lines 95% limits of agreement and error bars 95% confidence intervals.

10. Supplementary Figure 5

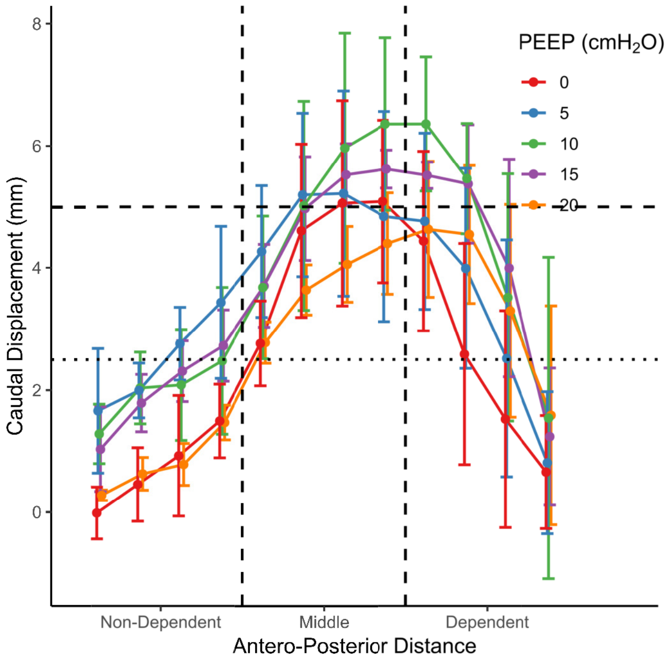

Supplementary Figure 5. Movement of a single 5 mm juxtadiaphragmatic CT slice of lung between end-expiratory and end-inspiratory breath holds at different PEEP levels. Tidal volume was fixed at 10 mL/kg in each case. The greatest caudal displacement was noted within the middle third of the lung in all cases. Dotted horizontal line represents a caudal displacement of 2.5 mm and dashed line 5 mm, equivalent to 50% and 100% of the slice moving out of the field-of-view of the CT scanner respectively. The latter condition was observed within the middle region at PEEPs of 0, 5, 10 and 15 cmH_2O and dependent region at PEEP 10 and 15 cmH_2O, and the displacement was equivalent to the adjoining slice moving into the scan field. Points represent mean and SD of n=4.

11. Supplementary Figure 6

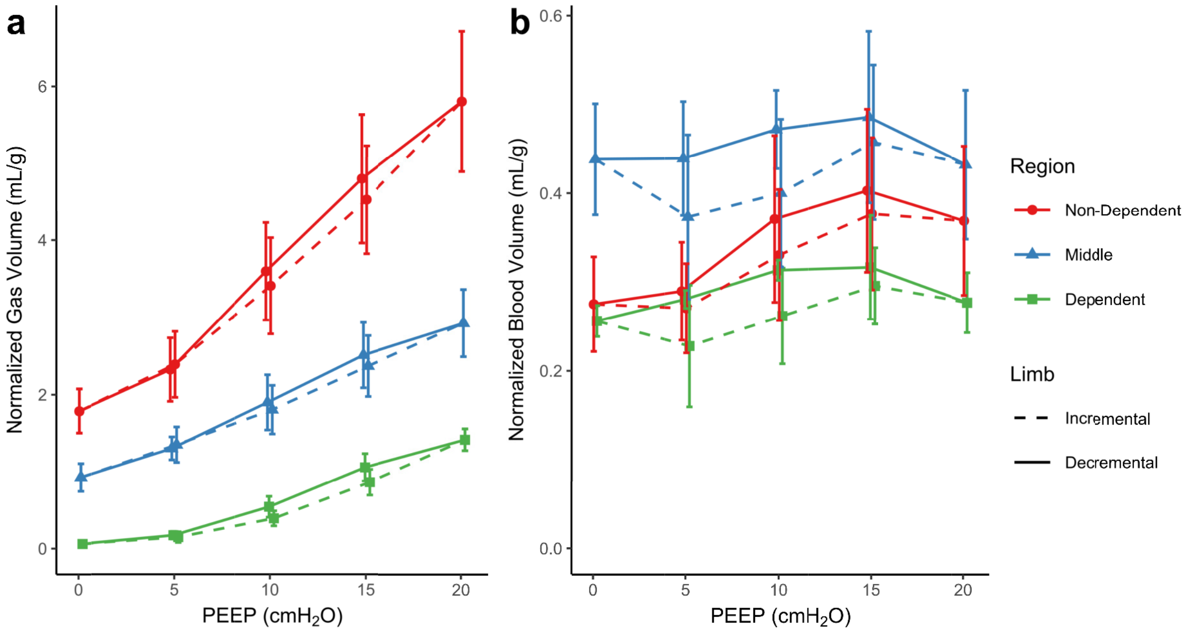

Supplementary Figure 6. Absolute values for gas (a) and blood (b) volumes normalized to lung tissue mass during expiration across all PEEP levels on both the incremental and decremental limbs of the PEEP trial within three gravitational regions. Classical hysteresis curves were observed for normalized gas volumes with the greatest aeration seen in the non-dependent region. The greatest values for normalized blood volume were observed in the middle region, and blood volume was relatively preserved across the range of PEEP values. Points represent mean and SD.

12. Supplementary Figure 7

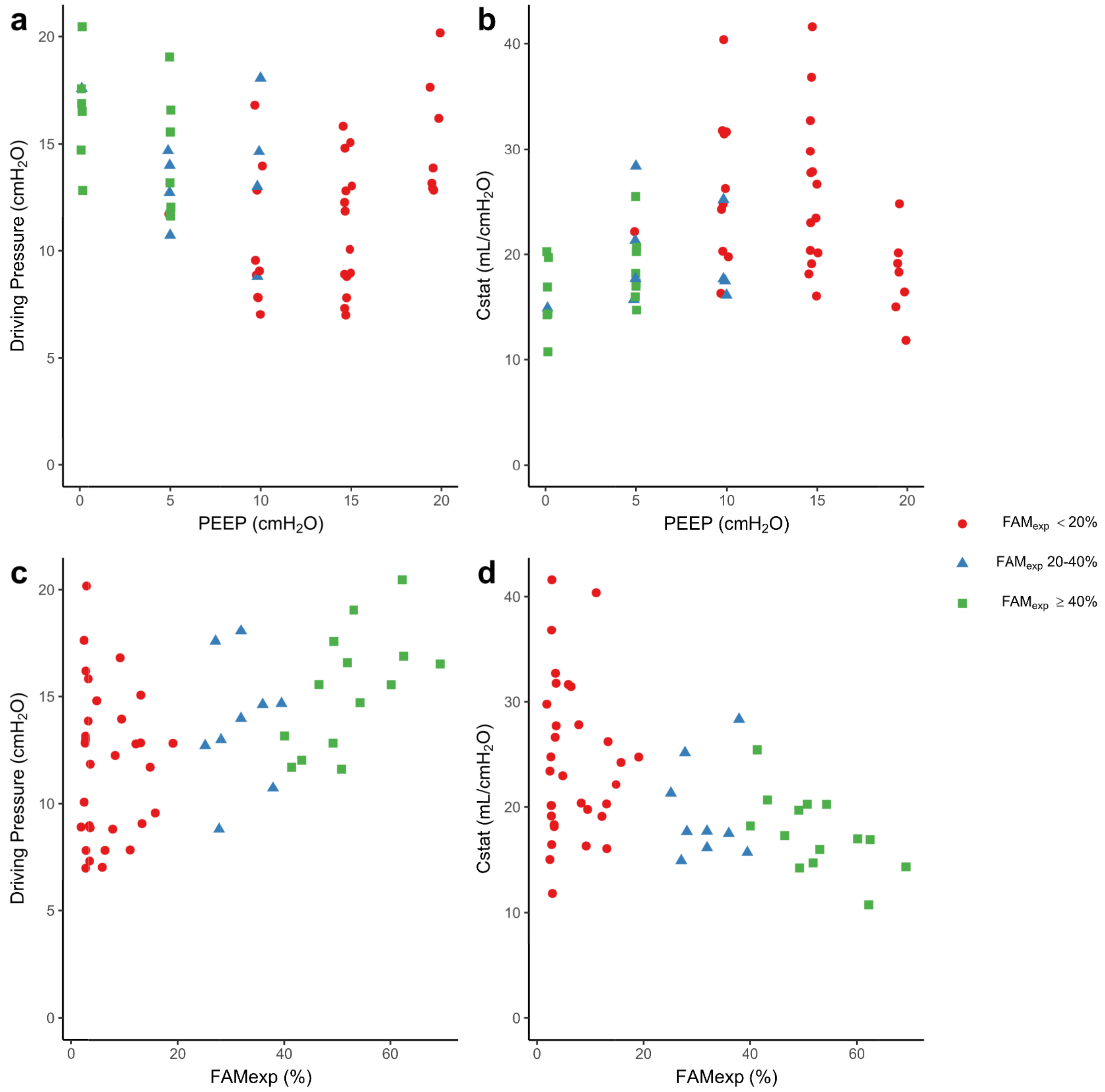

Supplementary Figure 7. Driving pressures (a,c) and compliance (b,d) throughout the PEEP trial (a,b) and expressed against fraction of the mass of the lung that was atelectatic in expiration (FAMexp; c,d). Each point represents a single ventilatory condition per animal. Driving pressure calculated as the difference between plateau pressure and PEEP and static compliance (Cstat) as the ratio of tidal volume and driving pressure.

13. References

1. Lachmann B, Robertson B, Vogel J. In vivo lung lavage as an experimental model of the respiratory distress syndrome. Acta Anaesthesiol Scand 1980;24(3):231-236.
2. Bourin M, Jolliet P, Ballereau F. An overview of the clinical pharmacokinetics of x-ray contrast media. Clinical pharmacokinetics 1997;32(3):180-193.
3. Cronin JN. Dual-energy computed tomography (DECT) three material differentiation algorithm. Available from: https://github.com/jncronin/dect
4. Fedorov A, Beichel R, Kalpathy-Cramer J, et al. 3D Slicer as an image computing platform for the quantitative imaging network. Magn Reson Imaging 2012;30(9):1323-1341.
5. Gattinoni L, Pesenti A, Bombino M, et al. Relationships between lung computed tomographic density, gas exchange, and PEEP in acute respiratory failure. Anesthesiology 1988;69(6):824-832.
6. Bland JM, Altman DG. Statistical methods for assessing agreement between two methods of clinical measurement. Lancet 1986;1(8476):307-310.
7. Modat M, Ridgway GR, Taylor ZA, et al. Fast free-form deformation using graphics processing units. Comput Methods Programs Biomed 2010;98(3):278-284.
8. Hurtado DE, Villarroel N, Andrade C, et al. Spatial patterns and frequency distributions of regional deformation in the healthy human lung. Biomech Model Mechanobiol 2017;16(4):1413-1423.
9. Hurtado DE, Villarroel N, Retamal J, et al. Improving the accuracy of registration-based biomechanical analysis: a finite element approach to lung regional strain quantification. IEEE Trans Med Imaging 2016;35(2):580-588.
10. Holm S. A simple sequentially rejective multiple test procedure. Scand J Stat 1979;6(2):65-70.
11. R Core Team. R: A language and environment for statistical computing. Vienna, Austria: R Foundation for Statistical Computing; 2016.
